# The Effect of Lockdown on Physiological Parameters of Sleep, Diet, Bowel Movement, Physical Activity, and Menstrual Cycle of Individuals Residing in Northeast India

**DOI:** 10.7759/cureus.81651

**Published:** 2025-04-03

**Authors:** Rituparna Barooah, Shakthinag S., Zakiyyah Tasneem, Jayshree Phurailatpam, Karabi Baruah, Anupi Das, Kahua Das, Gitartha Bordoloi, Tazkira Begum, Ranjana Dhar, Naveen P., Biakhlupuii Chhakchhuak, Shib Sekhar Datta, Kodumuri Praveen Kumar, John A Lyngdoh, Deisha B Rymbui, Iohborlang Rymbai

**Affiliations:** 1 Physiology, North Eastern Indira Gandhi Regional Institute of Health and Medical Sciences (NEIGRIHMS), Shillong, IND; 2 Physiology, Regional Institute of Medical Sciences (RIMS), Imphal, IND; 3 Physiology, Tezpur Medical College, Tezpur, IND; 4 Physiology, Jorhat Medical College, Jorhat, IND; 5 Physiology, Nagaon Medical College and Hospital, Nagaon, IND; 6 Physiology, Kokrajhar Medical College, Kokrajhar, IND; 7 Physiology, Assam Medical College, Dibrugarh, IND; 8 Physiology, Silchar Medical College, Silchar, IND; 9 Physiology, All India Institute of Medical Sciences (AIIMS), Guwahati, IND; 10 Physiology, Zoram Medical College, Aizawl, IND; 11 Community Medicine, Tripura Medical College and Dr. BRAM Teaching Hospital, Agartala, IND; 12 Physiology, Tomo Riba Institute of Health and Medical Sciences, Naharlagun, IND

**Keywords:** circadian rhythm, covid 19 impact of lockdown, diet pattern, lockdown, post-lockdown, sleep quantity

## Abstract

Background

Lockdown is a state of confinement that results in changes to the environmental factors that otherwise help entrain to circadian rhythm, and hence the need arises to understand the changes to various physiological factors during lockdown.

Methodology

A cross-sectional questionnaire-based study was conducted using snowball sampling, including people residing in Northeast India. A chi-square test was applied for the association between variables. A two-tailed Z test was done to analyze significant changes between two proportions.

Result

The final sample size was 992, and the mean age was 27.38 ± 10.887 years. A significant delay in bedtime (*P* = 0.0271), an increase in sleep latency (*P* = 0.0083), screen time duration (*P* = 0.0293), daytime sleep (*P* = 0.0155), and restfulness of sleep (*P* < 0.00001), as well as a decrease in sleep without awakening (*P* = 0.0001), were observed during lockdown compared to before the lockdown. In subjects sleeping beyond 1 AM, a significant decrease in restfulness (*P* = 0.00084) and an increase in sleep latency of more than one hour (*P* = 0.00528) were observed. An increase in water intake (508, 52%), fruit juice consumption (452, 46.1%), vegetable consumption (516, 52.9%), snacking (515, 52.8%), and body weight (447, 46.3%), along with a decrease in junk food consumption (527, 54.5%), were observed during lockdown. A significant decrease in alcohol consumption was observed during (*P* = 0.00338) and after (*P* = 0.03572) the lockdown. Breakfast timing was delayed in 395 individuals (40.5%), with a significant delay noted in those sleeping beyond 1 AM (*P* = 0.00634). A significant decrease in the frequency of lower stool passage (*P* = 0.01314) and a reduction in formed stools (*P* = 0.01016), along with a decrease in morning defecation (*P* = 0.0001), were observed. A significant decrease in morning defecation was observed in those sleeping beyond 1 AM (*P* = 0.01208). The changes in restfulness, sleep disturbance, screen time duration, sleep latency, defecation timing, and alcohol consumption observed during lockdown persisted even after the lockdown.

Conclusions

The most significant changes were seen for sleep. Changes in diet were inclined toward positive health. Sleeping beyond 1 AM, however, predisposed to longer latency of sleep, reduction in restfulness of sleep, and delayed breakfast and defecation time. Some of the changes observed during lockdown persisted even after the relaxation of restrictions.

## Introduction

*Lockdown* is an emergency protocol that prevents the public from moving from one area to another. Although concepts of isolation have been used during the Plague of Justinian (527-565 CE), it was in the 14th century that lockdown was documented to be employed in modern times [1]. It can be used as a preventive measure as well as an emergency plan to preserve the lives of those who are vulnerable or at risk [2].

As an aftermath of the COVID-19 pandemic, lockdown was extended in various phases for almost 1.5-2 years. During the lockdown, everyone except the frontline workers were confined to their homes, each of them holding an enormous amount of anxiety and anticipation for reasons starting from the unavailability of basic resources, the fight to survive, the overload of information and misinformation through the media handles about rising morbidity and mortality due to COVID-19, to the inability to put this free time to productive use as a result of restrictions due to social confinement. Many people experienced slight to drastic changes in their lifestyle and habits, including diet, physical activity, and sleep, which ultimately led to various changes in the physiological functioning of the body. This survey aimed to analyze the changes people reported in their habits and lifestyle, directly or indirectly, as a result of the lockdown and determine whether there was a meaningful parallel reflection in physiological parameters - if so, assessing the magnitude of the impact. It is a humble attempt to evaluate the statement made by Hall et al.: "The world will recover from the COVID-19 pandemic and so-called normal activities will resume; however, the 'Physical inactivity and sedentary behavior pandemic' will continue and, more troublingly, we may be at risk for this pandemic to worsen as a result of COVID-19" [3].

## Materials and methods

Study design

This research was conducted as a cross-sectional study.

Aims

To describe the changes in physiological parameters before, during, and after the lockdown in individuals residing in Northeastern India. 

Study population

The study population included individuals living in the Northeastern states of India during the 2020 lockdown, aged ≥16 years. Subjects who developed post-COVID-19 sequelae, had a history of neuropsychiatric illness, or were taking medications for sleep were not included in the study.

Study measures

Data were collected through a survey, and the questionnaire comprised closed-ended questions (Appendices A-F) distributed online (in English) via Google Forms or as a hard-copy version translated into Khasi, Assamese, or Bengali for local participants. This was conducted from September 2020 to March 2021, with responses filled out retrospectively for the first lockdown in India.

Timeline of lockdown in India in 2020

The lockdown in India was implemented from March 24 to May 31, 2020. The period before March 24 was considered before lockdown, while the period from March 24 to May 31 was considered during lockdown. The relaxation of lockdown occurred in six phases from June to November 2020. This period was considered after lockdown.

Sampling method

The questionnaires (Appendices A-F) were distributed to staff, workers, and students of institutes of the investigators, which included medical colleges from Northeast India, and they, in turn, forwarded it further; thus, a non-probability snowball sampling was performed.

Ethics statement

Informed consent was included at the start of the survey. The study was approved by the North Eastern Indira Gandhi Regional Institute of Health and Medical Sciences (NEIGRIHMS) Ethics Committee (Approval No: P137/2020/137).

Variables

Based on the variables for which information was sought, the questionnaire was divided into six sections: Appendix A: Sociodemographic (name, age, sex, address, status of living during lockdown); Appendix B: Sleep (bedtime, time taken to fall asleep, last activity before sleep, duration of last activity before sleep, sleep disturbances, restfulness of sleep, duration of daytime sleep); Appendix C: Physical Activity (duration, timing); Appendix D: Diet (snacking, coffee, junk food, water intake, fruit juice, vegetarian intake, appetite, body weight, alcohol consumption, meal timing of breakfast, lunch, dinner); Appendix E: Bowel (stool frequency, consistency, defecation time); Appendix F: Menstrual Cycle (cycle length, flow, symptoms of dysmenorrhea).

Statistical analysis

Categorical variables were expressed in proportion and graphs, while continuous variables were expressed in mean and standard deviation. The chi-square test was applied for determining the association between variables. A two-tailed Z-test was used to analyze significant changes between two proportions. Significance levels were taken as a *P*-value of 0.05.

## Results

Sociodemographic profiles

A total of 1,017 responses were collected, of which 25 were excluded due to inadequate data. The final sample size included was 992. The descriptive statistics for the sociodemographic profile are given in Table 1. The mean age of the participants was 27.38 ± 10.89 years. Twenty subjects did not report their age. The majority of participants belonged to the 20-29 age group. All the 992 subjects were residing in Northeast Indian states during the lockdown. The majority of subjects included in the study were originally from Northeast India (929, 93.5%), of whom 543 (54.7%) belonged to Assam and 133 (13.4%) to Meghalaya. The majority of subjects were also living with their parents during the lockdown (884, 89.1%).

**Table 1:** Sociodemographic profiles. Percentages may not sum to exactly 100% due to rounding to two decimal places.

Group	*n* (%)
Age group (in years)	
16-19	96 (9.68%)
20-29	589 (59.38%)
30-39	157 (15.3%)
40-49	83 (8.37%)
50-59	33 (3.31%)
≥60	14 (1.41%)
Unreported	20 (2.02%)
Gender	
Female	455 (45.9%)
Male	522 (52.6%)
Unreported	15 (1.5%)
Living with during the lockdown	
With family	884 (89.1%)
Alone	81 (8.2%)
With friends	19 (1.9%)
Unreported	8 (0.8%)
State belonging to	
Assam	543 (54.7%)
Meghalaya	133 (13.4%)
Manipur	124 (12.5%)
Arunachal	55 (5.54%)
Tripura	40 (4.03%)
Nagaland	19 (1.91%)
Mizoram	15 (1.51%)
Other	46 (4.63%)
Unreported	17 (1.71%)

To improve compliance with the questionnaire, response options were not made mandatory, resulting in incomplete data for some variables. Only subjects who provided a complete set of responses for a variable before, during, and after the lockdown were included in the analysis. The number of samples for each variable was as follows: bedtime (918), time taken to fall asleep (948), last activity before sleep (931), duration of last activity before sleep (933), disturbance to sleep (923), restfulness (957), daytime sleep (840), stool frequency (920), defecation time (916), stool consistency (918), alcohol consumption (770), duration of physical activity (887), timing of physical activity (824), menstrual cycle length (356), menstrual flow (356), and dysmenorrhea (369).

**Figure 1:**
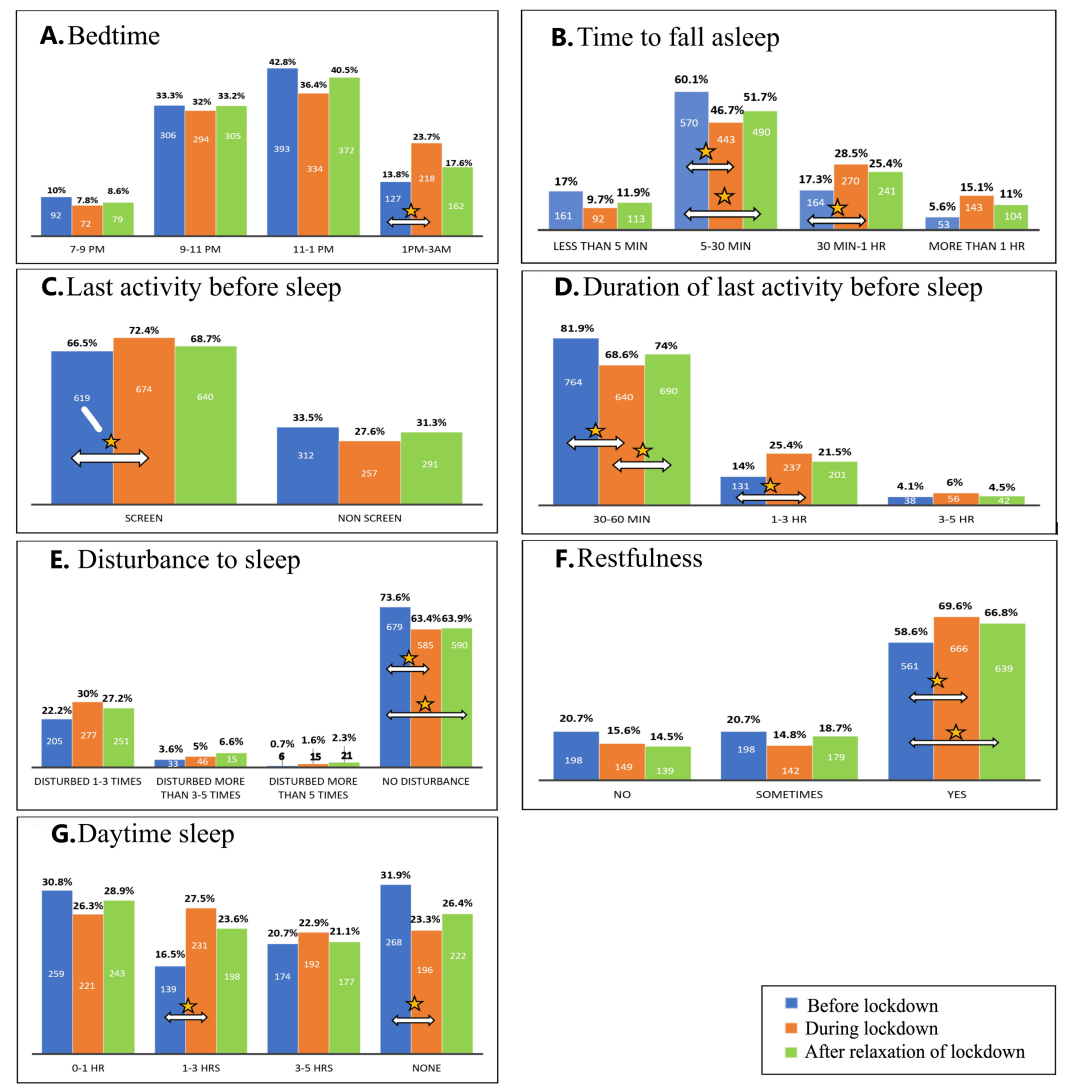
Reported proportions for sleep-related variables before, during, and after the lockdown. (A) A significant increase in the proportion of subjects with a bedtime between 1 AM and 3 AM was observed during the lockdown compared to before the lockdown (*P* = 0.0271, *z* = -2.2148). (B) A significant decrease in the proportion of subjects with a sleep latency of 5-30 minutes occurred during (*P* < 0.00001, *z* = -4.2466) and after (*P* < 0.00001, *z* = 4.2466) the lockdown compared to before. Additionally, a significant increase in those taking 30 minutes to 1 hour to fall asleep was seen during the lockdown (*P* = 0.0083, *z* = -2.6389). (C) A significant increase in screen time as the last activity before sleep was observed during the lockdown compared to before the lockdown (*P* = 0.02144, *z* = -2.3035). (D) The proportion of subjects spending a shorter duration (<60 minutes) on the last activity before sleep significantly decreased during (*P* < 0.00001, *z* = 5.7982) and after (*P* = 0.00028, *z* = 3.6403) the lockdown compared to before the lockdown. However, despite this decrease, the same duration significantly increased after compared to during the lockdown (*P* = 0.02926, *z* = -2.1775). Additionally, more subjects engaged in a longer activity duration (1-3 hours) before sleep during the lockdown than before (*P* = 0.02926, *z* = -2.1775). (E) A significant decrease in the proportion of subjects reporting no disturbances was observed during (*P* = 0.0001, *z* = 9.054) and after (*P* = 0.0002, *z* = 3.7295) the lockdown compared to before the lockdown. (F) A significant increase in the restfulness of sleep was observed during (*P* < 0.00001, *z* = -4.0132) and after (*P* = 0.00338, *z* = -2.935) the lockdown compared to before the lockdown. (G) A significant increase in daytime sleep duration of 1-3 hours was observed during the lockdown compared to before (*P* = 0.01552, *z* = -2.4215). Additionally, there was a significant decrease in the proportion of subjects reporting no daytime sleep during the lockdown compared to before (*P* = 0.04236, *z* = 2.0321).

Data from Figure 1 suggest a shift to a later bedtime, increased sleep latency, greater sleep disturbances, and more daytime sleep during lockdown. However, perceived restfulness of sleep was higher during and after the lockdown. There was also an increase in screen time before sleep during the lockdown. 

An increase in the intake of water, fruit juice, and a vegetarian diet, along with a decrease in the intake of junk food during the lockdown, can be seen. Higher tendencies toward snacking and an increase in body weight were also seen during the lockdown. The majority of subjects reported unchanged coffee intake and appetite during the lockdown (Table 2). A large proportion also reported delayed breakfast (Table 3).

**Table 2:** Descriptive statistics of dietary and related behavioral changes during the lockdown compared to before.

	Decreased, *n* (%)	Increased, *n* (%)	Same as before, *n* (%)
Snacking	124 (12.7%)	515 (52.8%)	336 (34.5%)
Coffee	229 (24.1%)	204 (21.5%)	516 (54.4%)
Junk food	527 (54.5%)	167 (17.3%)	273 (28.2%)
Water intake	71 (7.3%)	508 (52%)	397 (40.7%)
Fruit juice	185 (18.9%)	452 (46.1%)	343 (35%)
Veg intake	100 (10.3%)	516 (52.9%)	359 (36.8%)
Appetite	166 (16.89%)	382 (38.86%)	435 (44.25%)
Body weight	171 (17.7%)	447 (46.3%)	348 (36%)

**Table 3:** Descriptive statistics of meal timing during the lockdown compared to before.

	At the same time, *n *(%)	Delayed, *n *(%)	Earlier, *n *(%)	No regular timing, *n *(%)
Breakfast	406 (41.6%)	395 (40.5%)	49 (5%)	125 (12.8%)
Lunch	489 (51.3%)	208 (21.8%)	112 (11.7%)	145 (15.2%)
Dinner	512 (53.8%)	185 (19.4%)	128 (13.4%)	127 (13.3%)

**Figure 2:**
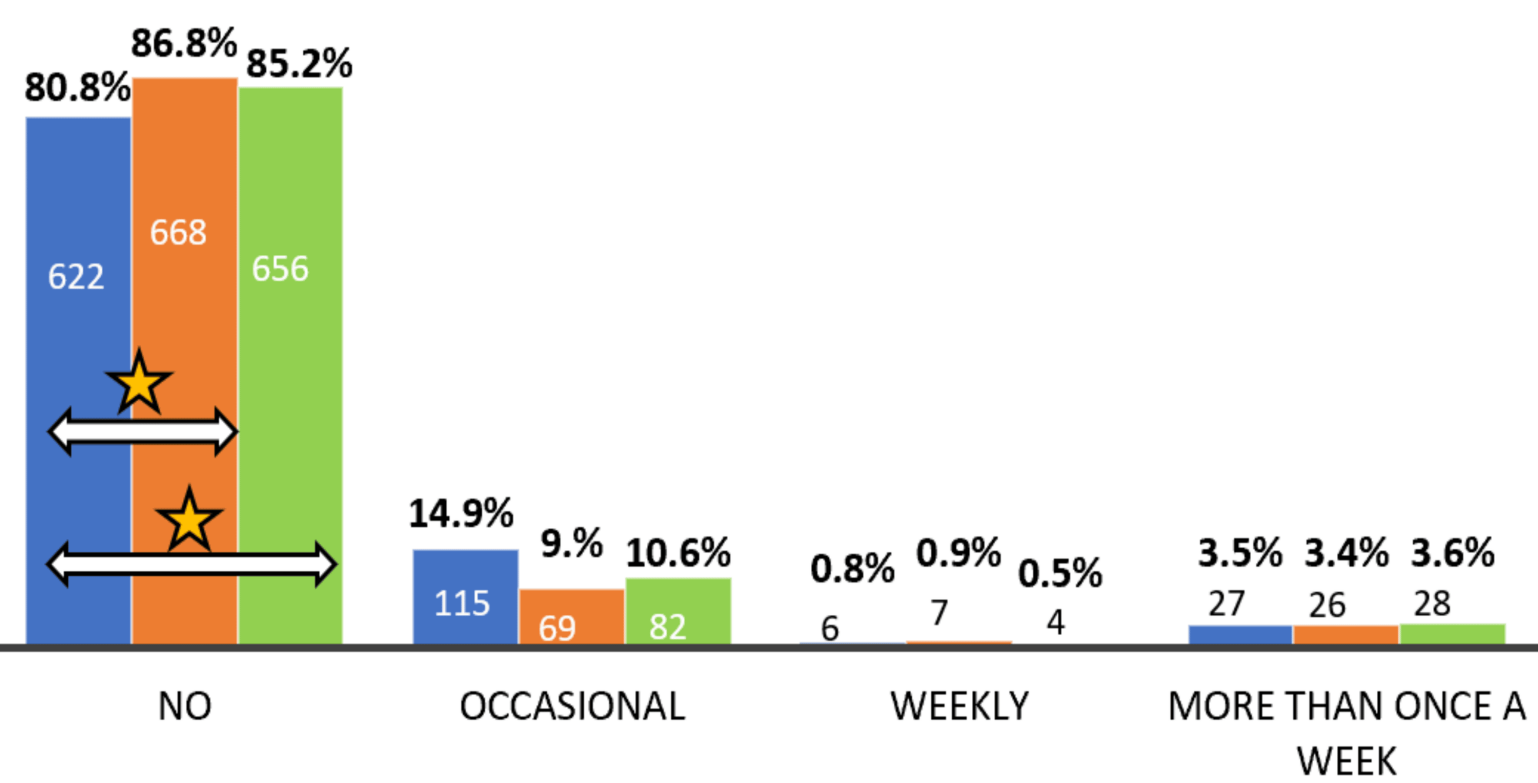
Proportion of alcohol consumption in subjects before, during, and after the lockdown. The star symbol indicates a significant change, while the arrow symbol denotes the analyzed pair showing significant differences. A significant increase in subjects reporting no alcohol consumption was observed during the lockdown compared to before (*P* = 0.00338, *z* = -2.9304). This trend persisted after the lockdown, with a significantly higher proportion of subjects reporting no alcohol consumption compared to before (*P* = 0.03572, *z* = -2.0959).

**Figure 3:**
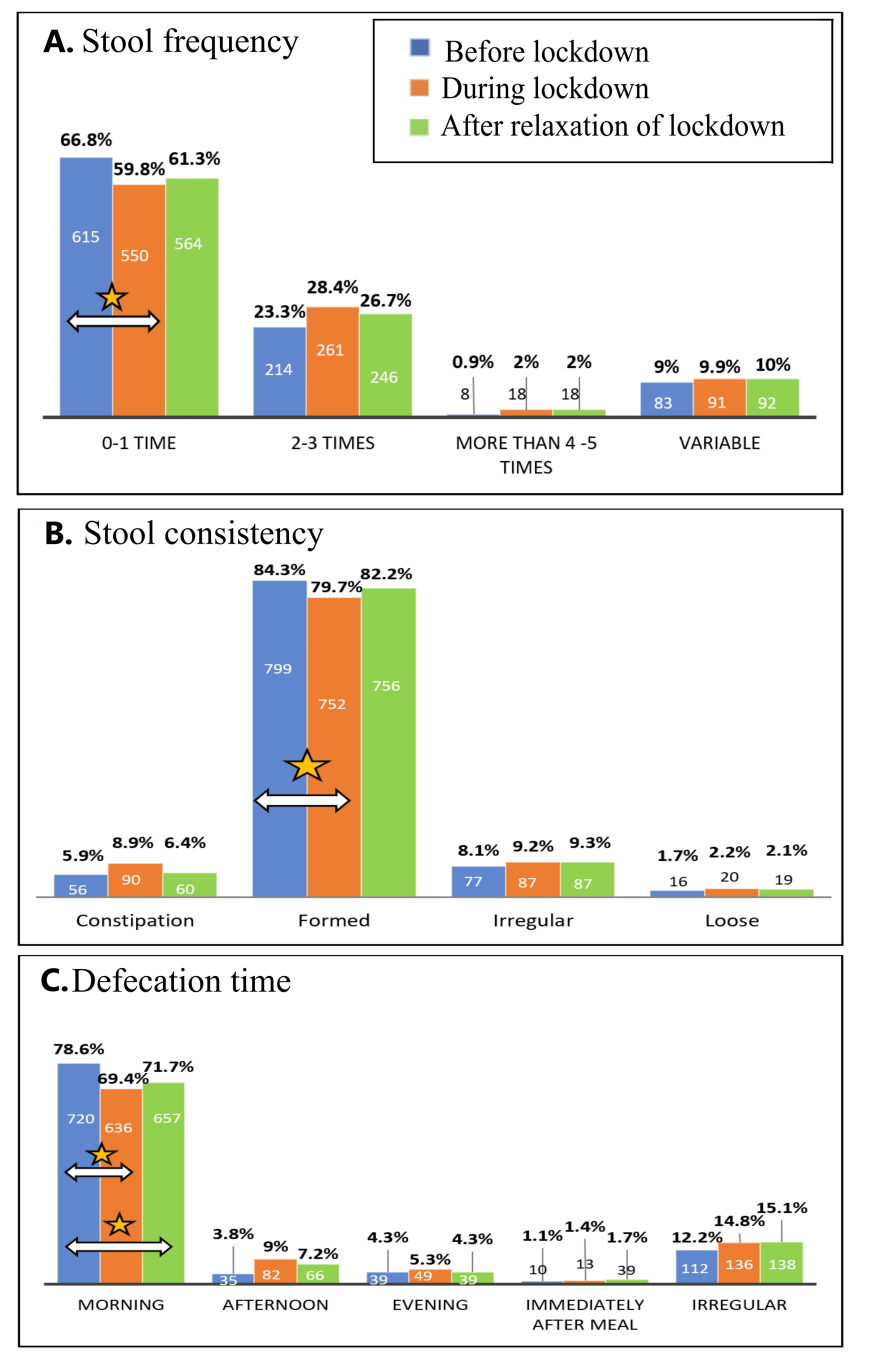
Reported proportions of variables related to bowel movements before, during, and after the lockdown. The star symbol indicates a significant change, while the arrow symbol denotes the analyzed pair showing significant differences. (A) A significant decrease in stool frequency (0-1 time) was observed during the lockdown compared to before (*P* = 0.01314, *z* = 2.4775). (B) A significant decrease in formed stools was observed during the lockdown compared to before (*P* = 0.01016, *z* = 2.573). (C) A significant reduction in subjects reporting morning defecation was seen during (*P* = 0.0001, *z* = 3.8682) and after (*P* = 0.00298, *z* = 2.9657) the lockdown compared to before.

A significant decrease in alcohol intake was seen during the lockdown. The number of individuals reporting abstinence from alcohol increased significantly and persisted even after the lockdown (Figure 2). During the lockdown, morning defecation significantly decreased and shifted to later times, persisting even after the lockdown. A significant reduction in formed stools and lower stool frequency was also observed (Figure 3).

No significant changes were observed in the duration or timing of physical activity, as well as in menstrual cycle parameters, including length, flow, and symptoms of dysmenorrhea, across the periods before, during, and after the lockdown.

**Figure 4:**
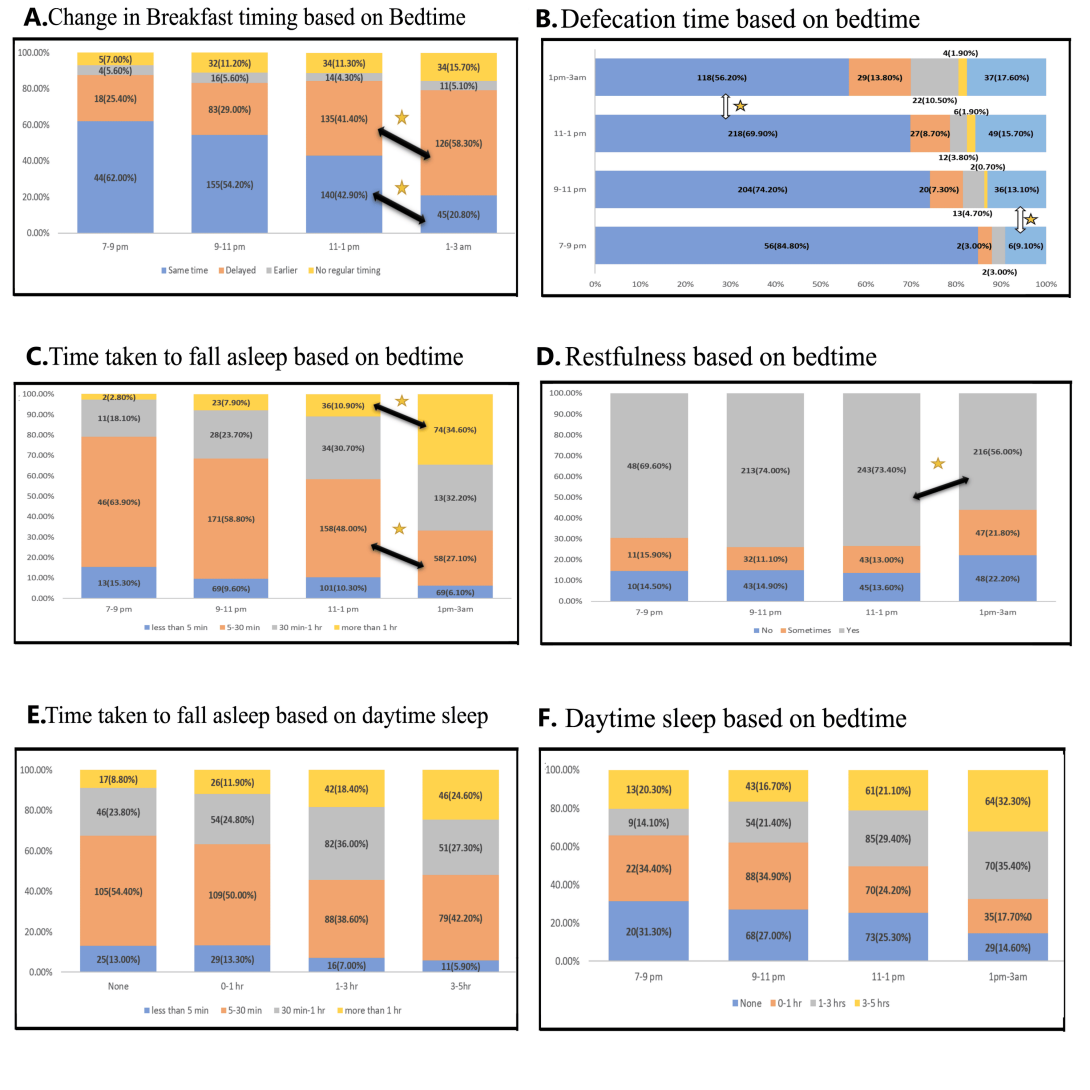
Proportions of variables showing significant association based on the chi-square test. The star symbol indicates a significant change, while the arrow symbol denotes the analyzed pair showing significant differences. (A) A significant increase in delayed breakfast was observed in subjects with a bedtime of 1-3 AM compared to 11 PM to 1 AM (*P* = 0.00634, *z* = -2.7288). Additionally, the proportion of subjects reporting no change in breakfast timing significantly decreased among those sleeping at 1-3 AM compared to 11 PM-1 AM (*P* = 0.0008, *z* = 3.3547). (B) A significant decrease in the proportion of subjects reporting morning defecation was observed in those with a bedtime of 1-3 AM compared to 11 PM-1 AM (*P* = 0.01208, *z* = 2.5147). Additionally, a significant increase in the proportion of subjects reporting irregular defecation times was seen in individuals sleeping beyond 9 PM compared to those with earlier bedtimes (7-9 PM) (*P* < 0.00001, *z* = 4.1231). (C) A significant increase in sleep latency of >1 hour was observed in subjects with a bedtime of 1-3 AM compared to 11 PM-1 AM (*P* = 0.00528, *z* = -2.7932). Additionally, a significant decrease in shorter sleep latency (5-30 minutes) was seen in subjects with a bedtime of 1-3 AM compared to 11 PM-1 AM (*P* = 0.00596, *z* = 2.7548). (D) A significant decrease in restfulness of sleep was observed in subjects sleeping at 1-3 AM compared to 11 PM-1 AM (*P* = 0.00084, *z* = 3.342). (E) Although the proportion of individuals taking longer to fall asleep increased with longer daytime sleep duration, no significant changes were observed in pairwise analysis between proportions. (F) Later bedtimes were associated with a higher proportion of individuals having longer daytime sleep. However, no significant changes were observed in pairwise analysis between proportions.

A chi-squared test was conducted to assess the association between variables during the lockdown, as reported in Table 4. Latency of sleep, restfulness of sleep, daytime sleep, change in breakfast timing, defecation time, and duration of physical activity showed a significant association with bedtime. Additionally, latency of sleep was associated with daytime sleep. Further pairwise analysis for associated variables is presented in Figure 4. No significant changes were seen when a pairwise analysis was done for bedtime and physical activity.

**Table 4:** Results of the chi-square test for association between variables. df, degrees of freedom

	n	df	*P*-value
Change in breakfast timing based on bedtime	899	9	0.000*
Defecation time based on bedtime	863	12	0.003*
Time taken to fall asleep based on bedtime	906	9	0.000*
Restfulness based on bedtime	904	6	0.000*
Time taken to fall asleep based on the type of activity before sleep	910	2	0.630
Time taken to fall asleep based on daytime sleep	826	9	0.000*
Daytime sleep based on bedtime	803	9	0.000*
Time to fall asleep based on duration of physical activity	912	9	0.099
Bedtime based on duration of physical activity	890	9	0.029*
Disturbance of sleep based on duration of physical activity	916	9	0.308
Bedtime before and during the lockdown	918	9	0.000*

It can be inferred from Figure 4 that subjects with later bedtimes experienced an increase in delayed breakfast and later defecation times. They also had longer sleep latency and decreased restfulness. No significant changes were observed in daytime sleep based on bedtime or in sleep latency based on daytime sleep (Figure 4).

Although a significant association was found between bedtime before and during the lockdown (Table 4), no significant changes were observed using the two-tailed Z-test for proportions. Descriptive statistics are provided in Table 5. Notably, 58.7% of individuals who slept later than 1 AM during the lockdown had a bedtime between 11 PM and 1 AM before the lockdown.

**Table 5:** Comparison between bedtime before and during the lockdown.

Bedtime		During lockdown			
		7-9 PM	9-11 PM	11 AM-1 PM	1-3 AM
Before the lockdown	7-9 PM	60 (83.30%)	5 (6.90%)	4 (5.60%)	3 (4.20%)
	9-11 PM	28 (9.50%)	204 (69.40%)	54 (18.40%)	8 (2.70%)
	11 PM-1 AM	4 (1.20%)	83 (24.90%)	207 (62.00%)	40 (12.00%)
	1-3 AM	0 (0.00%)	14 (6.40%)	128 (58.70%)	76 (34.90%)

A total of 42.1% of participants who had not exercised before the lockdown reported engaging in 30 minutes to 1 hour of exercise during the lockdown, while those who exercised previously continued their routine (Table 6).

**Table 6:** Comparison between the duration of physical activity before and during the lockdown.

Physical activity		During lockdown			
		0 hour	30 minutes-1 hour	1-2 hour	>2 hours
Before lockdown	0 hour	128 (49.40%)	109 (42.10%)	20 (7.70%)	2 (0.80%)
	30 minutes-1 hour	77 (17.20%)	286 (64.00%)	72 (16.10%)	12 (2.70%)
	1-2 hours	33 (28.70%)	33 (28.70%)	42 (36.50%)	7 (6.10%)
	>2 hours	8 (11.90%)	15 (22.40%)	16 (23.90%)	28 (41.80%)

## Discussion

Sleep

Bedtime

A delay in bedtime was seen during the lockdown, and our study found a statistically significant rise in the number of people who slept past 1:00 AM (Figure 1A). Previous studies from different parts of the world conveyed a similar picture, showing a delay in bedtime during the lockdown [4-8]. Most studies compared data from before and during the lockdown but not after. In this study, the percentage of bedtime returned to near pre-lockdown levels after the relaxation of lockdown (Figure 1A).

The bedtime before lockdown was associated with bedtime during lockdown (Table 4), and subjects who slept later than 11 PM before lockdown had a higher likelihood of having later bedtimes during lockdown (Table 5). Further research is needed to fully understand the innate tendencies of late chronotypes during lockdown, when they are not constrained by social norms. This is important because later bedtimes have been associated with poorer long-term health outcomes, depressive states, and lower sleep quality [9].

Time to Fall Asleep/Latency

During the lockdown, the study observed an increase in sleep latency (Figure 1B). This aligns with a previous study [10] conducted in South India among 310 pharmacy students, which also reported a longer time required to fall asleep during the lockdown.

It is also seen in our study that the changes to latency of sleep persisted even after lockdown, unlike changes in bedtime, which returned to normal (Figure 1B), and this latency was greater for later bedtimes (Figure 4C). This could be because bedtime is influenced by sociocultural factors such as work and school, which could be the reason why they returned to normal after the lockdown was lifted. It could be that the changes to the circadian rhythm and sleep-wake cycle might take a longer time to come back to the normal rhythm after being entrained to environmental factors. Further prospective studies should be done to ascertain how long it takes for lockdown-imposed changes for latency of sleep to return back to normal levels, as the risk of bias might be present in retrospective questionnaires.

Disturbance to Sleep

The number of subjects reporting having no disturbance to sleep had a significant reduction during lockdown, which persisted after lockdown (Figure 1E). In previous studies, especially in young adults and females, an increase in disturbed sleep (periods of awakening) has been reported [6,11]. Stress, changes in lifestyle and circadian rhythm, apprehensions about the virus, and lack of social interactions could all be contributing factors. There was, however, no association with physical activity (Table 4). Future studies need to identify the factors for these changes.

Daytime Sleep

Longer daytime sleep durations were observed in the study during lockdown (Figure 1G). This is consistent with other research that found that during the lockdown, there was an increase in either daytime drowsiness or daytime naps [4,5]. Although another study highlighted that in teenagers, as opposed to young adults, an improvement in daytime sleepiness was reported during the lockdown [6]. It could also be because younger individuals had more structured sleep enforced by parents. Since the study found that they slept longer and woke slightly later than usual school hours, their need for daytime sleep was reduced. In contrast, older individuals without such restrictions tended to sleep later, reducing sleep restfulness and increasing the need for daytime sleep catch-up.

Restfulness

A significantly higher percentage of subjects reported having a restful sleep during the lockdown, and this persisted even after the lockdown (Figure 1F). A previous study showed an improvement in sleep quality, particularly among adolescents, due to increased sleep duration [6,12]. Although we did not report sleep duration, this could explain the increased restfulness observed during lockdown in individuals who went to bed earlier.

However, other studies have reported a decrease in sleep quality during lockdown, with evening-type chronotypes being the most affected [13]. Our findings align with this, as individuals sleeping between 1 and 3 AM reported a significant reduction in sleep restfulness compared to those with earlier bedtimes (Figure 4D). A recent study highlights that bedtimes beyond 1 AM, regardless of chronotype, are associated with poorer mental health outcomes, with a reduction in REM sleep duration being one of the proposed mechanisms for this [14]. This reduction in REM sleep could also explain the difference in the subjective feeling of restfulness between those sleeping early and late.

Last Activity Before Sleep

Despite the lack of comparative data, studies indicate that screen time increased during lockdown [15-17]. Our findings on the significant rise in screen time during lockdown align with previous studies (Figures 1C, 1D). However, the time taken to fall asleep did not differ significantly based on whether individuals used screens or engaged in other activities before sleep (Table 4), contradicting the majority of reports [18-20]. It should also be noted that the sample size for individuals with screen use (*n *= 674) was much larger than those without (*n *= 257). A prior study demonstrates that only an increase in screen time was associated with an increase in sleep latency, while those who maintained or reduced their screen time experienced no changes [21]. Additionally, a Brazilian study reveals that the negative association between screen time and sleep is exclusive to people who lead sedentary lives [19]. Thus, it's possible that the relationship between screen time and sleep is not as linear as anticipated and that there are a number of other factors to take into account.

Diet

We observed an increase in the intake of water, fruit juice, and a vegetarian diet, along with a decrease in junk food consumption during the lockdown. However, snacking behaviors increased, accompanied by weight gain (Table 2). Regarding meal timing, 395 (40.5%) of individuals reported a delay in breakfast timing (Table 3). There was also a significant association between breakfast timing and bedtime (Table 4), with a significant delay in breakfast time among those who slept past 1 AM. A previous study reported that late chronotypes are more likely to experience delays in meal timings, with breakfast being the most affected [22].

There are mixed findings in literature pertaining to changes in dietary habits during lockdown. A study in Brazil [23] found results that were in contrast to ours, showing a rise in the consumption of fast food, bakery goods, and instant meals, while there was a reduction in the consumption of fruits and vegetables. Their behavior of snacking tended to be of higher frequency in the evening, with lower snacking during breakfast, morning, and lunch hours. In our study, although an increase in snacking was observed, we have not specified the distinction of the timing of the snacking habit. The pattern of later bedtime along with delayed breakfast that we have found in our study could also be a reason for this evening predilection for snacking.

Another study in Columbia [24] also showed an increase in the consumption of the so-called *comfort foods* (cariogenic) like candies, filled doughnuts, toffees, and sugar-sweetened bubble gums. It was determined that certain factors, such as being single, having children, and being under lockdown, heightened this tendency.

A study in Italy [25] also reported a slight increase in the intake of fruits and vegetables, similar to the findings of our study. However, in contrast to the decline in fast food observed in our study, they also reported excessive consumption of comfort food, sweets, or pastries. However, we did not specify the definition of junk food, which could introduce bias due to the subjective variability of what constitutes junk food.

A study in Spain [26] indicated that the majority of the sample maintained their diets as before lockdown, and those who did change mostly leaned toward more healthy dietary habits (similar to our findings), like increasing vegetables and fruits and decreasing alcohol and industrial pastries.

The heterogeneity of results in the literature highlights that geographical and cultural influences affect changes in dietary habits during lockdown. The majority of our study population consists of subjects from northeastern India (929, 93.5%), who have a distinct dietary pattern compared to the rest of India, with a greater reliance on green leafy vegetables.

There was a significant decrease in alcohol intake during lockdown, with a notable increase in the number of individuals reporting abstinence from alcohol, along with a reduction in occasional drinkers, though the latter was not statistically significant (Figure 2). This trend persisted even after the lockdown. This could be due to the social restrictions imposed by the lockdown, a decrease in availability of alcohol, or the fear of disease transmission through occasions, which would predispose someone to transmission. It could also be due to the fact that the majority of subjects in our study were living with their families during the lockdown. As a previous study reported, living alone was a factor associated with increased alcohol consumption during strict lockdowns [27]. It should also be noted that individuals who consumed alcohol at least weekly before the lockdown did not show any significant changes during the lockdown. A similar finding has been reported, with 60.6% of drinkers experiencing no change in consumption [28] and 90.4% reporting no change in both alcohol and smoking status [29].

Previous studies on alcohol consumption report mixed results, with some showing a decrease [27, 30, 31] and others indicating an increase [23,32-35]. Factors associated with increased alcohol consumption include younger age, having more children at home, COVID-19-related unemployment, being a non-health worker, working from home, household stress, urban living, and living alone [28,32].

Bowel movements

An increase in frequency and a reduction in consistency were seen in this study (Figure 3). There was also a shift from defecating in the morning to later in the day, and this habit persisted even after the lockdown. This shift in defecation time could partly be attributed to delayed awakening, as subjects who slept past 1 AM showed a significant reduction in morning defecation (Figure 4B).

A study in Mexico reported a 25% new-onset incidence of constipation during lockdown, with these subjects experiencing a significant decrease in stool frequency and consistency [36]. In our study, however, there was a 3% increase in reported constipation during lockdown, which was not statistically significant. They also report that in subjects without constipation, there were no significant changes to stool frequency or consistency, unlike the findings of our study. These changes might be linked to previously discussed differences in dietary habits.

There have also been reports of altered gut microbiota due to changes in diet, sleep patterns, and stress levels, which are known to correlate with symptoms of anxiety and depression [37].

Physical activity

This study found no evidence of any significant differences in the timing or duration of physical activity before, during, or after the lockdown's relaxation. Previous studies have mixed findings with both reduction and increase in physical activity reported. It should also be noted that we have not made any distinction regarding types or grades of physical activity.

According to a Scottish study [38], both sitting time and moderate-to-intense physical activity increased during lockdown, while walking decreased. After the relaxation of lockdown, activity levels returned to pre-lockdown levels.

A different study conducted in Italy and Poland [39,40] found that during lockdown, children and adolescents showed a decrease in physical activity and an increase in sedentary behavior. A decline in physical activity was also reported by a study conducted in Malaysia and China [41,42].

In our study, 109 (42.1%) of the participants who had not exercised before the lockdown reported exercising for at least 30 minutes to an hour during the lockdown, while those who had exercised previously continued to do so (Table 6). This could be due to the initiation of home workout routines as part of the influence of increasing screen time and social media.

However, a study in Spain found opposing results, reporting that lockdown had a greater impact on physically active individuals, leading to a significant decline in their physical activity, while physically inactive individuals showed no significant changes [43].

A study in Germany showed that among the elderly, the number of individuals engaged in intense activities decreased, and two-thirds of the participants failed to resume them after the second lockdown. Regarding resistance exercise, performance increased during the first lockdown but declined during the second. The authors postulate that this could be due to the prolonged effects of lockdown or seasonal variation, as the second lockdown occurred during the winter period [44].

A significant association was found between bedtime and the duration of physical activity (Table 4). This aligns with another study reporting that a shorter duration of physical activity was associated with poorer sleep [45]. However, sleep latency and sleep disturbances showed no association with physical activity in our study (Table 4).

Menstrual cycle

There were no significant changes in the menstrual cycle concerning cycle length, flow, or the occurrence of dysmenorrhea. Although there was an increase in the number of individuals with scanty flow (that persisted after lockdown), it was not statistically significant.

Psychological stress, diet, physical activity, and circadian rhythm are factors that can influence female reproductive health during lockdown. While existing literature discusses the effects of COVID-19 infection [46,47] and the COVID-19 vaccine [48-51] on menstrual cycle alterations, limited research is available on the impact of lockdown itself. Moreover, the studies that do exist show significant heterogeneity in their results [52].

Strengths and limitations

The study has the following strengths that it compares the changes in physiological parameters from before, during and after the relaxation of lockdown, providing a longitudinal axis to the findings. It is also the first study of the kind in the Northeastern Indian population.

The study has a few limitations. The questionnaire was filled out retrospectively and might be at risk of recall bias (Appendices A-F). The questionnaire used was not validated, although previous studies used similar questions. Due to incomplete responses, slightly different samples were used for each variable analysis. As a result, the demographic profile of the entire sample may not accurately reflect the demographics of each variable analysis.

Due to the higher proportion of individuals belonging to the age group less than 30 years, the results might not reflect the entire population. Although major changes were seen during sleep, including the duration of sleep and the midsleep point, they could have better reflected the chronotype of the individual and added more strength to the study. There might be multiple other factors involved that we have not considered in the study that could affect these parameters, like viral load, infection to relatives and friends, quarantine and isolation, financial constraints, and pre-existing disturbances to sleep and mood.

## Conclusions

The study helps elucidate the effects of lockdown on various physiological profiles, with the most pronounced changes observed in sleep parameters. This was more specific for people sleeping later than 1 AM during the lockdown. Although screen time increased, it was not associated with changes in sleep latency. Changes in sleep patterns during the lockdown led to a shift toward later mealtimes and defecation times. Changes in diet patterns were also seen, which was more reflective of better health in this population compared to previous studies in other parts of the world. No significant changes were seen in physical activity and menstrual cycle. Although the findings of later sleep habit, delayed breakfast timing, and bowel habits follow a logical progression of events, it is interesting to note that the changes in sleep latency and defecation time persisted even after lockdown relaxation, despite the bedtime reverting to near normal. This calls for the importance of formulating guidelines and mitigation strategies while imposing lockdowns, taking into consideration the physiological changes it brings about. The changes in restfulness, sleep disturbance, screen time duration, sleep latency, defecation time, and alcohol consumption observed during lockdown persisted even after restrictions were lifted. Future prospective studies are needed to determine the time required for these changes to revert.

## Appendices

Appendix A

**Table 7:** Sociodemographic Please tick against the correct box under the options and fill in the blanks if required

Sr No	Question	Option	Tick
	Sex	Male	
		Female	
		Prefer not say	
2	Age		
3	Describe your stay during lockdown.	Alone	
		With family	
		With friends	
4	Which state do you belong to?	Assam	
		Meghalaya	
		Mizoram	
		Manipur	
		Nagaland	
		Arunachal Pradesh	
		Tripura	
		Other	

Appendix B

**Table 8:** Questionnaire on sleep Please tick against the correct box under the options and fill in the blanks if required

This section will be related to questions related to your Sleep.				
What was your average bedtime during the mentioned periods?				
	7 – 9 pm	9 – 11 pm	11pm – 1 am	1am – 3am
Before lockdown				
During Lockdown				
After Lockdown				
How long does it take for you to fall asleep after going to bed?				
	Less than 5 min	5 - 30 min	30min – 1 hr	More than 1hr
Before lockdown				
During Lockdown				
After Lockdown				
Do you have any disturbance to sleep during the periods mentioned?				
	No disturbance	Disturbed 1-3 times	Disturbed 3-5 times	Disturbed more than 5 times
Before lockdown				
During Lockdown				
After Lockdown				
How would you respond to the statement, ‘I feel well rested with the sleep’?				
	Yes	No	Sometimes	
Before lockdown				
During Lockdown				
After Lockdown				
How much time do you spend sleeping during daytime?				
	None	0-1hr	1-3hr	3-5hr
Before lockdown				
During Lockdown				
After Lockdown				
How would you describe your last activity before sleep on an average basis?				
	Screen based	Not screen based		
Before lockdown				
During Lockdown				
After Lockdown				
How long would you spend on the above-mentioned activity usually before bedtime?				
	30 – 60 min	1 – 3hr	3 – 5 hr	
Before lockdown				
During Lockdown				
After Lockdown				

Appendix C

**Table 9:** Questionnaire on Physical activity Please tick against the correct box under the options and fill in the blanks if required

This section will be related to questions related to your Physical activity.					
How long did you usually spend engaging in physical activity during the mentioned periods?					
	0 hr	30min – 1 hr	1 – 2 hr	More than 2hr	
Before lockdown					
During Lockdown					
After Lockdown					
What were your usual timings for the above-mentioned physical activity?					
	Morning	Afternoon	Evening	Night	No fixed time
Before lockdown					
During Lockdown					
After Lockdown					

Appendix D

**Table 10:** Questionnaire on diet Please tick against the correct box under the options and fill in the blanks if required

This section will have questions enquiring about your Diet habits during the mentioned periods.				
How has your intake of coffee been in the mentioned periods?				
	Increased	Decreased	Same as before	
Before lockdown				
During Lockdown				
After Lockdown				
How has the intake of junk food been in the mentioned periods?				
	Increased	Decreased	Same as before	
Before lockdown				
During Lockdown				
After Lockdown				
How has the intake of water been in the mentioned periods?				
	Increased	Decreased	Same as before	
Before lockdown				
During Lockdown				
After Lockdown				
How has the intake of fruit juices been in the mentioned periods?				
	Increased	Decreased	Same as before	
Before lockdown				
During Lockdown				
After Lockdown				
How has the intake of vegetables been in the mentioned periods?				
	Increased	Decreased	Same as before	
Before lockdown				
During Lockdown				
After Lockdown				
How would you describe your appetite in the mentioned periods?				
	Increased	Decreased	Same as before	
Before lockdown				
During Lockdown				
After Lockdown				
How has your frequency of snacking been during the mentioned periods?				
	Increased	Decreased	Same as before	
Before lockdown				
During Lockdown				
After Lockdown				
How has your bodyweight been in the mentioned periods?				
	Increased	Decreased	Same as before	
Before lockdown				
During Lockdown				
After Lockdown				
How would you best describe the timing of your breakfast during the mentioned period?				
	At the same time	Delayed	Earlier	No regular timing
Before lockdown				
During Lockdown				
After Lockdown				
How would you best describe the timing of your lunch during the mentioned period?				
	At the same time	Delayed	Earlier	No regular timing
Before lockdown				
During Lockdown				
After Lockdown				
How would you best describe the timing of your dinner during the mentioned period?				
	At the same time	Delayed	Earlier	No regular timing
Before lockdown				
During Lockdown				
After Lockdown				
How often did you consume alcohol during the mentioned period?				
	No	Occasional	Weekly	More than once a week
Before lockdown				
During Lockdown				
After Lockdown				

Appendix E

**Table 11:** Questionnaire on bowel habits Please tick against the correct box under the options and fill in the blanks if required

This section will be asking you about questions related to your Bowel habits					
What would be the time that you most often defecate?					
	Morning	Afternoon	Evening	Irregular	
Before lockdown					
During Lockdown					
After Lockdown					
How many times a day do you defecate?					
	0 – 1 time	2 – 3 times	More than 4 – 5 times	Variable	
Before lockdown					
During Lockdown					
After Lockdown					
How would you describe the consistency of your stool?					
	Formed	Constipation	Loose	Irregular	
Before lockdown					
During Lockdown					
After Lockdown					

Appendix F

**Table 12:** Questionnaire on menstrual cycle Please tick against the correct box under the options and fill in the blanks if required

This section is for women and has questions related to your Menstrual history				
How would you describe your Menstrual flow during the mentioned periods?				
	Normal	Heavy	Scanty	
Before lockdown				
During Lockdown				
After Lockdown				
Do you find your menstruation painful?				
	Yes, always	No	Sometimes	
Before lockdown				
During Lockdown				
After Lockdown				
How would you describe your average Menstrual cycle?				
	Regular cycle 28-30 days	Regular cycle 31-35 days	Irregular	
Before lockdown				
During Lockdown				
After Lockdown				
